# A framework for microalgal biosorption of heavy metals: mechanisms, modeling, and critical insights

**DOI:** 10.1039/d6ra05731d

**Published:** 2026-08-18

**Authors:** Heba M. Youssef, Mohamed S. Abd Elhameed, Fatma Mohamed

**Affiliations:** a Botany and Microbiology Department, Faculty of Science, Beni-Suef University 62511 Egypt hebamahmoud926@gmail.com +20-10-2805-6093; b Nanophotonics and Applications Lab, Faculty of Science, Beni-Suef University Beni-Suef 62514 Egypt; c Materials Science Research Laboratory, Chemistry Department, Faculty of Science, Beni-Suef University Beni-Suef Egypt

## Abstract

Heavy metal contamination of aquatic systems poses serious risks to human health and the environment, requiring efficient and sustainable treatment strategies. Owing to the abundance of functional groups, high surface reactivity, and structural plasticity of microalgal biomass, microalgal biosorption has emerged as a promising approach for heavy metal remediation. This review provides a comprehensive and critical evaluation of microalgal biosorption, focusing on its mechanistic basis, comparative performance, environmental and operational controls, biomass diversity, and modeling approaches. Major physicochemical processes governing heavy metal uptake, including ion exchange, surface complexation, electrostatic interactions, and precipitation, are systematically discussed, with an emphasis on the roles of surface functional groups and biomass characteristics. The effects of key operational parameters, including pH, temperature, ionic strength, initial metal concentration, contact time, competing ions, and biomass state, are critically evaluated, highlighting their interactive effects on biosorption performance. Comparative analysis further demonstrates that biosorption efficiency is highly system-dependent and varies with the target metal, microalgal biomass, wastewater composition, and treatment configuration. Commonly applied kinetic, isotherm, and thermodynamic models are critically examined, revealing their limitations in representing heterogeneous biomass surfaces, multimetal competition, mass-transfer, and complex real wastewater matrices. Advanced spectroscopic and theoretical methods, including XPS, XAS/EXAFS, and density functional theory, are further considered to clarify metal-specific coordination environments and the energetic basis of molecular interactions. A unified conceptual framework linking interfacial mechanisms, environmental and operational drivers, biomass diversity, and model-based interpretations is proposed. The review identifies the need to move beyond isolated parameter optimization and capacity-centered evaluation toward mechanism-informed and predictive biosorption frameworks supported by standardized experimentation, realistic wastewater validation, and systematic assessment of regeneration, scalability, techno-economic feasibility, and environmental sustainability.

## Abstract 2

This review integrates microalgal biosorption mechanisms, biomass diversity, environmental controls, and modeling to support predictive and sustainable heavy-metal remediation in realistic wastewater.

## Graphical abstract

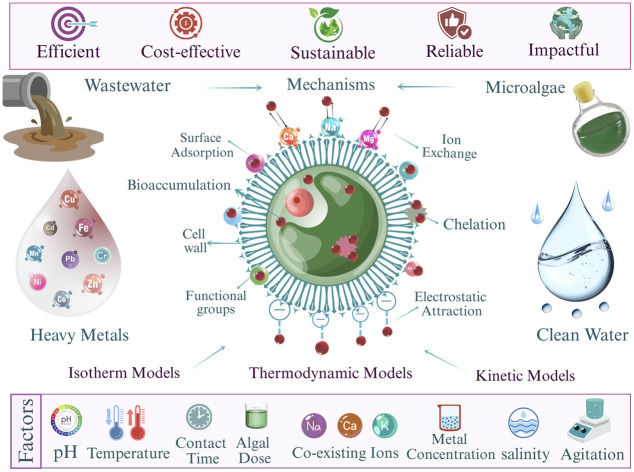

## 1. Introduction

Heavy metal contamination in aquatic environments has become one of the most critical and persistent environmental challenges worldwide, posing severe risks to ecosystems and all forms of life.^1^ This widespread pollution originates from both natural and anthropogenic sources, including mining activities, industrial effluents, ore processing, improper waste disposal, and rapid urbanization.^2^ Heavy metals are elements with a density greater than 5 g cm^−3^.^3^ Both essential and non-essential metals occurring naturally or originating from anthropogenic sources are among these contaminants.^4^ Maintaining the optimal balance of trace metals in biological systems is crucial for sustaining cellular function and enhancing productivity. Despite their indispensable role in physiological processes, elevated concentrations of these metals can induce severe toxicity in living organisms.^5^

Industrial processes such as tanning, electroplating, mining, metallurgy, chemical manufacturing, and battery production are among the major sources of heavy metal pollution.^6^ Furthermore, competitive interactions among metal ions may influence the ecological impacts of mixed-metal systems.^7^ Exposure to heavy metals has been associated with serious health effects, even at low concentrations, including neurological damage, renal dysfunction, and carcinogenic, mutagenic, and teratogenic effects.^8^ Hence, these toxic heavy metals must be removed from wastewater before discharge.^9^ Heavy metals are frequently removed from wastewater using conventional techniques such as chemical precipitation, ion exchange, membrane filtration, flotation, coagulation–flocculation, and electrochemical procedures.^10^ In parallel, the development of advanced adsorbent materials, including graphene-based materials, has expanded the range of strategies available for environmental pollution remediation, pollutant adsorption, and water treatment.^11^ However, the production, cost, regeneration, and environmental sustainability of advanced synthetic adsorbents remain important considerations for their broader application.^12^

Due to their affordability and environmental sustainability, bioremediation techniques based on microorganisms such as bacteria, fungi, yeasts, and microalgae have attracted increasing attention as alternatives to these approaches.^13^ Microalgae-based systems have emerged as viable alternatives for heavy metal removal from wastewater and provide the basis for environmentally sustainable and effective phycoremediation technologies.^14^

Carboxyl, hydroxyl, phosphate, and amino groups are among the functional groups present in microalgal cell walls that enable metal ions to bind through various physicochemical interactions.^15^ Microalgal biomass represents an economical, sustainable, scalable, and efficient option for wastewater treatment because biosorption is a metabolism-independent process that enables rapid metal uptake through mechanisms such as ion exchange, complexation, electrostatic attraction, and surface precipitation. Microalgal biosorption systems have been the subject of extensive research; however, removal efficiency has been shown to differ greatly among studies.^16–19^ This disparity, driven by differences in operational conditions, biomass characteristics, and environmental parameters, produces a fragmented view of system performance.

Moreover, previous reviews have generally addressed individual aspects of microalgal heavy metal removal, such as adsorption mechanisms, operational conditions, biosorbent performance, or modeling approaches, without sufficiently integrating these dimensions into a unified framework that connects molecular–scale interactions, including metal-specific coordination and binding energetics, with biomass diversity, system-level behavior, real wastewater complexity, and predictive process development. Accordingly, this review aims to provide a critical and integrative analysis of microalgal heavy metal biosorption by connecting molecular and interfacial mechanisms with environmental and operational drivers, biomass characteristics, treatment configurations, and process-level performance. Unlike conventional reviews that primarily summarize adsorption mechanisms or reported removal efficiencies, this work examines how these dimensions interact to determine biosorption behavior and practical applicability. The review critically evaluates the limitations of conventional kinetic, equilibrium, and thermodynamic models in describing heterogeneous biomass surfaces, multimetal competition, transport limitations, and complex wastewater matrices. It further considers molecular-level evidence obtained through advanced spectroscopic techniques, including XPS and XAS/EXAFS, together with theoretical approaches such as density functional theory (DFT), to clarify metal-specific coordination environments, surface chemical states, and the energetic basis of metal-biomass interactions. In addition, different microalgal biomass formats and biosorbent configurations, including live, non-viable, immobilized, modified, and biofilm-based systems, are compared using multidimensional criteria related to capacity, selectivity, stability, regeneration, mass transfer, and operational feasibility. Building on these integrated insights, the review identifies the major barriers to transferring laboratory-scale findings to realistic and continuous treatment systems and proposes a transition toward mechanism-informed and predictive frameworks that integrate surface chemistry, metal speciation, biomass properties, transport behavior, environmental variability, and data-driven approaches. This perspective provides a distinct value addition by shifting the discussion from isolated performance comparisons toward an integrated, mechanistic, molecularly informed, and predictive understanding of microalgal biosorption for scalable and sustainable wastewater treatment.

## 2. Review methodology

This review was developed using a structured critical literature-search and synthesis approach to evaluate recent advances in microalgal biosorption for heavy-metal removal from wastewater. The literature search focused primarily on studies published between 2023 and 2026, reflecting recent developments in biosorption mechanisms, molecular-level characterization, biomass engineering, wastewater treatment configurations, adsorption modeling, predictive modeling, and sustainable resource-recovery strategies. Earlier publications were selectively included when they provided essential foundational knowledge or established mechanistic, spectroscopic, theoretical, or modeling concepts relevant to the interpretation of recent advances.

Relevant literature was identified through searches of Scopus, Web of Science, and Google Scholar using combinations of keywords related to microalgae, biosorption, heavy metals, wastewater treatment, adsorption mechanisms, surface complexation, metal–biomass interactions, advanced spectroscopy, XPS, XAS, EXAFS, density functional theory, kinetic and equilibrium modeling, thermodynamics, immobilization, biofilms, modified biomass, real wastewater, predictive modeling, and resource recovery. The search was designed to cover the biological, molecular, physicochemical, engineering, and modeling dimensions of microalgal biosorption.

The selected literature was organized and critically evaluated according to the major themes addressed in this review, including the mechanisms governing metal uptake; molecular-level evidence obtained through advanced spectroscopic and theoretical approaches; the effects of operational and environmental conditions; differences among biomass configurations; kinetic, equilibrium, thermodynamic, and predictive modeling approaches; performance under complex wastewater conditions; and the challenges associated with regeneration, reuse, scale-up, and sustainability. Studies were compared with consideration of the target metal, wastewater characteristics, biomass properties, treatment configuration, operating conditions, analytical and characterization methods, proposed mechanisms, modeling approaches, and practical limitations. Rather than presenting the literature as a collection of isolated study summaries, the available evidence was synthesized to identify common trends, sources of variability, mechanistic consistencies and uncertainties, limitations in current approaches, and areas where molecular-level understanding remains insufficient. Particular emphasis was placed on evaluating how spectroscopic evidence and theoretical calculations complement conventional adsorption models and experimental performance data by clarifying metal-specific coordination environments, surface chemical states, binding interactions, and the energetic basis of metal-biomass interactions. This structured critical approach provided the basis for integrating molecular–scale interactions, operational factors, biomass configuration, process performance, and predictive modeling, while also identifying research gaps and future directions for the development of more reliable, transferable, and sustainable microalgal biosorption systems.

## 3. Mechanistic basis of microalgal biosorption systems

**Fig. 1:**
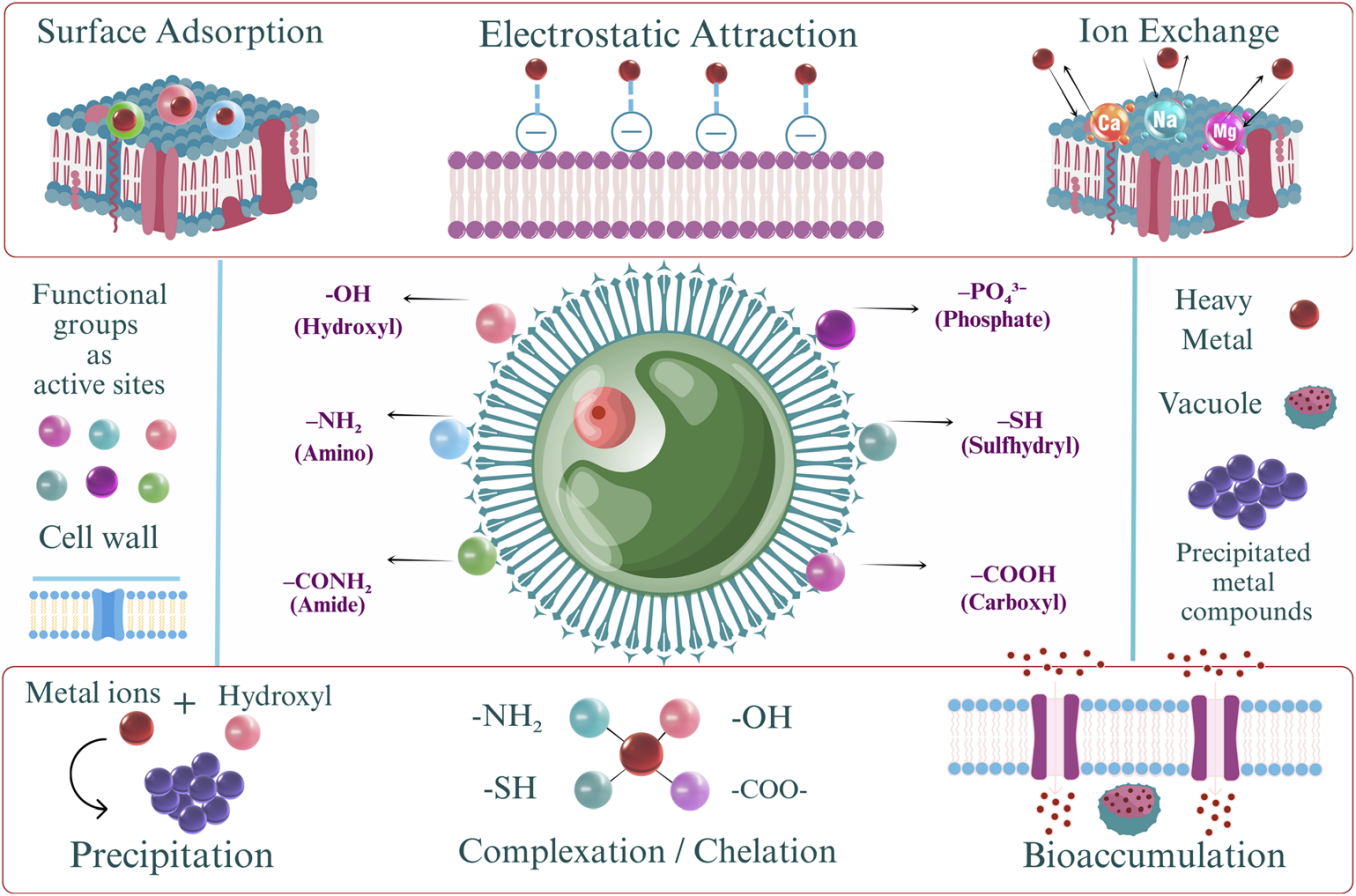
Comprehensive mechanisms of microalgal biosorption and metal uptake. Schematic overview of the principal mechanisms governing heavy metal removal by microalgae, including biosorption at the cell surface and interactions with cell-wall functional groups. Additional processes, such as ion exchange, electrostatic attraction, surface complexation, and intracellular sequestration, are depicted to illustrate the multimechanistic nature of microalgal metal removal systems. The figure emphasizes the coexistence and partial overlap of these mechanisms in living microalgal systems rather than assuming a strictly sequential or isolated pathway.

The microalgal biosorption system is governed by multiple physicochemical and biological mechanisms that determine metal uptake and partitioning between surface-bound and intracellular fractions, Fig. 1.

### 3.1. Biosorption and bioaccumulation: distinct pathways governing metal uptake

The physicochemical features of the microalgal cell surface, which acts as a dynamic and reactive interface, govern biosorption processes by enabling metal ions to passively bind to the surface without compromising cellular viability.^20^ This rapid and frequently reversible process is primarily driven by surface chemistry rather than intracellular metabolic activity. Consequently, both living and non-living biomass can serve as efficient biosorbents, providing operational stability, particularly under challenging wastewater conditions in which metabolic activity may be reduced.^21^ In contrast, bioaccumulation is an energy-dependent process in which metal ions are actively transported across the cell membrane before being sequestered in intracellular compartments.^22^ This process is mediated by membrane transport systems and intracellular detoxification mechanisms, including ligand complexation and organelle compartmentalization, making it highly dependent on cellular metabolic status.^23^ These fundamental differences explain why biosorption systems typically reach equilibrium more rapidly than processes involving intracellular metal uptake.^24^ However, in living microalgal systems, biosorption and bioaccumulation often occur simultaneously, making it difficult to distinguish their relative contributions. This overlap is further complicated by the lack of standardized analytical procedures for reliably distinguishing between surface-bound and intracellular metal fractions. Consequently, many studies report total metal removal without fractionation, which may overestimate biosorption capacity and introduce ambiguity into mechanistic interpretation.

Experimental studies have shown that heavy metal removal by microalgae such as *Dunaliella salina* and *Scenedesmus obliquus* involves both extracellular adsorption and intracellular accumulation, with biosorption representing the predominant mechanism.^25,26^ Nevertheless, the limited tolerance of microalgal cells can constrain bioaccumulation, as high metal concentrations may compromise membrane integrity, impair enzyme activity, and reduce overall metal uptake efficiency.^24^ Compared with bioaccumulation, which represents a distinct but more physiologically constrained detoxification pathway, biosorption is generally more reliable and operationally stable because it is independent of cellular metabolism.^27^ This duality highlights the need for integrated analytical approaches capable of distinguishing between these pathways to improve mechanistic understanding, reduce reporting bias, and support the development of scalable microalgal wastewater treatment systems.

### 3.2. Cell surface chemistry as a reactive interface (functional groups and binding sites)

To develop an effective microalgae-based tertiary treatment system for wastewater treatment plants, appropriate microalgal species must be selected. Different algal species possess distinct cell-wall compositions, resulting in differences in the distribution and abundance of functional groups.^28^ Consequently, even under identical operating conditions, the sensitivity and biosorption efficiency of microalgae may vary according to their genus and species.^23^ Several comparative studies have demonstrated substantial differences in heavy-metal removal efficiency among microalgal species.^29^ Additionally, the structure and surface chemistry of microalgal cell walls strongly influence biosorption efficiency, which can vary substantially among species. Because the cell wall represents a primary site of interaction between algal biomass and metal ions in aqueous solutions, its physicochemical characteristics directly influence metal-binding capacity and selectivity.^30^ Microalgal cell walls are composed of complex matrices of polysaccharides, glycoproteins, uronic acids, and, in some taxa, silica (*e.g.*, diatoms) or cellulose.^31^ These components contain a range of functional groups, including amino (–NH_2_), sulfate-containing (–SO_3_^−^), phosphate-containing (–PO_4_^3−^), carboxyl (–COOH), and hydroxyl (–OH) groups, which can promote complexation with positively charged metal ions and contribute to electrostatic interactions.^15^ Species-specific differences in the distribution, density, and accessibility of these functional groups influence metal affinity and uptake kinetics. For instance, the surface functional groups of *Chlorella* sp. may contribute to its affinity for metals such as Cd(ii), Pb(ii), and Cu(ii).^32^ Algal biomass can also be processed, for example, by drying, acid or base treatment, or chemical modification, to expose additional functional groups, alter surface structure, and improve sorption performance.^33^

### 3.3. EPS-mediated metal binding and mass transfer regulation

Metal sequestration is greatly facilitated by extracellular polymeric substances (EPS), in addition to the structural contribution of the cell wall.^34^ EPS contains numerous functional groups capable of binding and immobilizing metal ions, thereby forming a hydrated matrix around the cells. For example, functional groups such as hydroxyl (–OH), carboxyl (–COOH/–COO^−^), and carbonyl (C=O) groups play important roles in heavy-metal binding. Their involvement in metal complexation and ion binding has been demonstrated by shifts in, and the disappearance of, characteristic absorption bands following exposure to Cd(ii) and Pb(ii), as revealed by FTIR analyses of EPS.^35^ Importantly, EPS also influences mass-transfer processes by modifying diffusion resistance and generating additional binding domains outside the cell surface.^36^ For example, a recent study demonstrated that *Chlorella* sp. increased SOD activity and EPS production in response to Cd(ii) stress. Tryptophan-containing proteins-like substances within EPS, which is a protein-rich matrix, may also contribute to protection against Cd(ii) stress.^37^

### 3.4. Molecular and physicochemical mechanisms of metal removal

One of the principal processes underlying metal uptake in microalgal systems is ion exchange.^38^ Light metal ions associated with negatively charged functional groups on the cell surface can be exchanged for metal cations in solution during this process.^39^ Furthermore, because the availability of functional groups for metal binding is directly affected by their protonation and deprotonation states, this exchange process is strongly pH-dependent.^27^ Surface complexation and chelation are also important mechanisms in biosorption. For instance, functional groups such as amino, phosphate, and carboxyl moieties can form stable coordination bonds with metal ions, generating inner-sphere or outer-sphere complexes.^40^ The strength and specificity of these interactions depend on both the chemical properties of the metal ions and the structural characteristics of the binding sites.^22^ Furthermore, *Dunaliella salina* has been reported to possess specific functional groups, including –OH, –COOH, –NH, –NH_2_, P=O, and S=O, that contribute to heavy-metal removal through surface complexation and ion exchange.^25^ The initial attraction between metal ions and the microalgal surface is primarily driven by electrostatic interactions.^41^ Coulombic forces attract positively charged metal ions to negatively charged cell surfaces under appropriate pH conditions.^42^ By increasing the concentration of metal ions near the cell surface, these interactions promote subsequent binding processes, although they are typically weaker than chemical bonding. In addition to these processes, van der Waals forces and nonspecific interactions can contribute to physical adsorption.^43^ The biosorption process is further complicated by precipitation and redox transformations. In some circumstances, insoluble hydroxides or carbonates may precipitate on or near the cell surface.^34^ Heavy metals can influence biomineralization processes by inhibiting or altering calcium carbonate precipitation, whereas microalgal activity can promote calcite formation and contribute to metal removal through coprecipitation and adsorption onto mineral phases.^44^ Moreover, certain microalgae can indirectly alter redox conditions, thereby influencing metal speciation and mobility.^45^ Due to the overlap among these processes, it can be difficult to determine whether metal removal results from genuine biosorption or from chemically driven transformations occurring at the cell surface.^46^

Additionally, microalgal biosorption performance is governed by an interconnected network of surface interactions, including ion exchange, complexation, electrostatic attraction, and surface precipitation, rather than by a single mechanism. Environmental factors, including pH, ionic strength, and metal speciation, discussed in the following section, significantly influence the relative contribution of each mechanism.^47^ Overall, microalgal metal removal is therefore a multifaceted process arising from the interplay of several concurrent and often overlapping mechanisms that encompass surface interactions, extracellular processes, and intracellular uptake and transformation. Biosorption provides a rapid and largely metabolism-independent first barrier through the combined action of surface functional groups, ion exchange, electrostatic attraction, complexation, and extracellular polymeric substances, whereas bioaccumulation may contribute additional intracellular sequestration in metabolically active cells. However, the relative contribution of these pathways is strongly dependent on the physiological state of the biomass, cell-surface chemistry, extracellular polymeric matrices, metal speciation, and solution conditions. Accordingly, removal performance should not be interpreted as direct evidence of a specific mechanism unless surface-bound and intracellular metal fractions are experimentally distinguished and the potential contributions of precipitation and redox transformations are considered. This mechanistic distinction is essential for improving the comparability of studies and designing biosorption systems with predictable performance under realistic wastewater conditions.

### 3.5. Molecular-level mechanisms revealed by advanced spectroscopy and theoretical modeling

At the molecular level, advanced spectroscopic analyses have increasingly demonstrated that microalgal metal removal involves specific coordination environments rather than nonspecific surface accumulation. Conventional FTIR and XPS analyses can identify the functional groups involved in metal binding, whereas X-ray absorption spectroscopy (XAS) provides more direct information on the local coordination environment and chemical state of the bound metal. For example, spectroscopic analyses of *Cyanothece* sp. CE4 released polysaccharides revealed metal-specific coordination patterns: nickel preferentially coordinated with oxygen-containing donors, zinc showed a stronger association with chlorine-containing ligands, whereas copper exhibited less selective coordination. These observations demonstrate that the chemical identity of the metal ion influences the molecular configuration of biosorption complexes.^48^ Moreover, the coordination chemistry of the metal can also vary according to the biochemical fraction of the biosorbent and the presence of competing ions. In *Dactylococcopsis salina*, X-ray absorption and complementary spectroscopic analyses revealed distinct coordination patterns for Cu(ii), Ni(ii), and Zn(ii), with mixed O/N- and Cl/S-containing ligands contributing to metal binding.^49^ Importantly, the coordination environment was influenced by both the metal identity and multimetal exposure, directly linking biosorption chemistry with the subsequent catalytic performance of the metal-enriched biomass. This finding highlights that biosorption can generate chemically differentiated metal biomass complexes rather than a uniform pool of adsorbed ions.

Further molecular evidence has been obtained from XPS, FTIR, and XAS analyses of microalgal systems exposed to different metals. In *Chlamydomonas reinhardtii*, protein-derived carboxyl groups were primarily involved in Pb(ii) and Cd(ii) binding, whereas glycosidic bonds contributed predominantly to Cd(ii) and Zn(ii) interactions, indicating metal-specific contributions of extracellular polymeric functional groups.^50^ XPS and EXAFS analyses of uranium-exposed *Acutodesmus* sp. indicated that phosphate groups were primarily involved in uranium complexation, while micromorphological analyses revealed relatively uniform extracellular uranium distribution and heterogeneous intracellular accumulation patterns.^51^ In another study, the removal of Cu(ii), Cd(ii), and Zn(ii) by *Chlorella vulgaris* involved interactions with surface functional groups, together with metal-specific biomineralization processes, including the reduction of Cu(ii) to Cu(i) and the formation of precipitates containing copper, cadmium, and zinc.^52^ Collectively, these spectroscopic findings indicate that the molecular mechanism of microalgal biosorption is governed by metal-specific coordination, ligand selectivity, chemical speciation, and the spatial distribution of binding sites. Accordingly, FTIR peak shifts alone should not be interpreted as definitive evidence of a particular binding mechanism. A more robust mechanistic interpretation requires the integration of complementary techniques, particularly XPS for surface chemical states and XAS/EXAFS for local coordination environments, together with microscopic and elemental analyses. This integrated molecular-level approach provides a stronger basis for distinguishing surface complexation from precipitation, redox transformation, and intracellular sequestration, thereby improving the mechanistic reliability of microalgal biosorption studies.

The integration of theoretical calculations with experimental characterization has further advanced the molecular interpretation of algal-based sorption systems. Density functional theory (DFT) calculations showed that metal binding to algal-derived materials is governed by site-specific interactions rather than uniform surface adsorption. In algal biochar, DFT analyses identified complexation, ion exchange, and cation-π interactions as important contributors to metal binding, with a predicted affinity order of Cu(ii) > Fe(ii) > Zn(ii) and displacement of native cations such as K(i).^53^ Similarly, DFT analysis of algal organic matter revealed metal-dependent binding strengths, with the strongest interaction observed for Cu(ii), followed by Zn(ii) and Cd(ii).^54^ These findings demonstrate how theoretical calculations can complement spectroscopic measurements by linking experimentally observed metal coordination to the relative energetic stability of specific molecular interactions. However, such predictions should be interpreted as mechanistic support rather than a substitute for experimental validation, particularly because the chemical structure of algal biomass is highly heterogeneous and changes substantially after processing.

## 4. Comparative performance of microalgal biosorbents for heavy-metal removal

By harnessing the natural ecological functions of microalgae, including their high photosynthetic efficiency and relatively simple cellular organization, phycoremediation can effectively treat wastewater contaminated with heavy metals.^55^ Their ability to tolerate challenging environmental conditions, such as heavy-metal stress, nutrient limitation, high salinity, and temperature fluctuations, makes microalgae excellent candidates for environmental remediation applications. Phycoremediation has been reported as a cost-effective and time-efficient alternative to conventional biological and chemical treatment processes. Consistent with this concept, recent research has shown that microalgae are increasingly being employed for the removal of heavy metals from diverse wastewater sources, demonstrating their robust biosorption and bioremediation potential under a range of environmental conditions and contaminant types.^14,47^

A mixed culture of *Chlorella vulgaris* and *Scenedesmus obliquus* achieved high removal efficiencies for Cu(ii) (98.25–99.9%) and Fe(iii) (85.68–99.19%).^56^ Notably, *S. obliquus* showed greater tolerance to heavy-metal stress than *C. vulgaris*, whereas the use of mixed-culture and biofilm-based configurations improved system stability and metal uptake, highlighting the influence of biomass organization on microalgal biosorption performance.^56^ Similarly, green microalgae demonstrated substantial metal-removal performance in coal-mine wastewater, achieving removal efficiencies of 85% for Fe(iii), 95% for Zn(ii), 99% for Cd(ii), and 100% for Cu(ii) and Al(iii). These results highlight the potential of microalgal systems for treating metal-rich industrial effluents while also illustrating the metal-specific variability of removal performance.^57^ At an optimal pH of 7, dried *Chlorella sorokiniana* biomass exhibited a high Cd(ii) biosorption capacity of up to 92 mg g^−1^.^58^ However, the removal performance of microalgal biosorbents can vary substantially depending on the target metal and treatment configuration. For example, *Dunaliella* sp., AL1 achieved removal efficiencies of 20.11–26.67% for Cu(ii) and 94.99–95.51% for Cr(vi) in single- and bimetallic systems, respectively.^59^ Similarly, chitosan immobilization enabled microalgal biomass to achieve a Cr(vi) adsorption capacity of up to 37.1 mg g^−1^, highlighting the potential of immobilized configurations for improving the practical applicability of microalgal biosorbents.^60^ Beyond heavy-metal removal, microalgal wastewater treatment systems can simultaneously remove nutrients and metals, with reported removal efficiencies of up to 99.6% for nitrogen, 100% for phosphorus, and 13–100% for different heavy metals across diverse wastewater types.^61^ Overall, microalgal biosorption can achieve substantial heavy-metal removal across diverse metals and wastewater matrices, although reported removal efficiencies and adsorption capacities vary considerably among species and treatment systems. This variability reflects differences in metal identity, initial concentration, pH, biomass characteristics, cultivation and pretreatment conditions, as well as the use of single, mixed, biofilm, or immobilized systems. Consequently, no single microalgal species or treatment configuration consistently provides the highest performance across all metals and wastewater conditions. Biosorption efficiency is therefore strongly system-specific, highlighting the importance of matching the biological characteristics of the biomass and the treatment configuration to the target metal and wastewater matrix.

## 5. Environmental and operational controls on biosorption performance

**Fig. 2:**
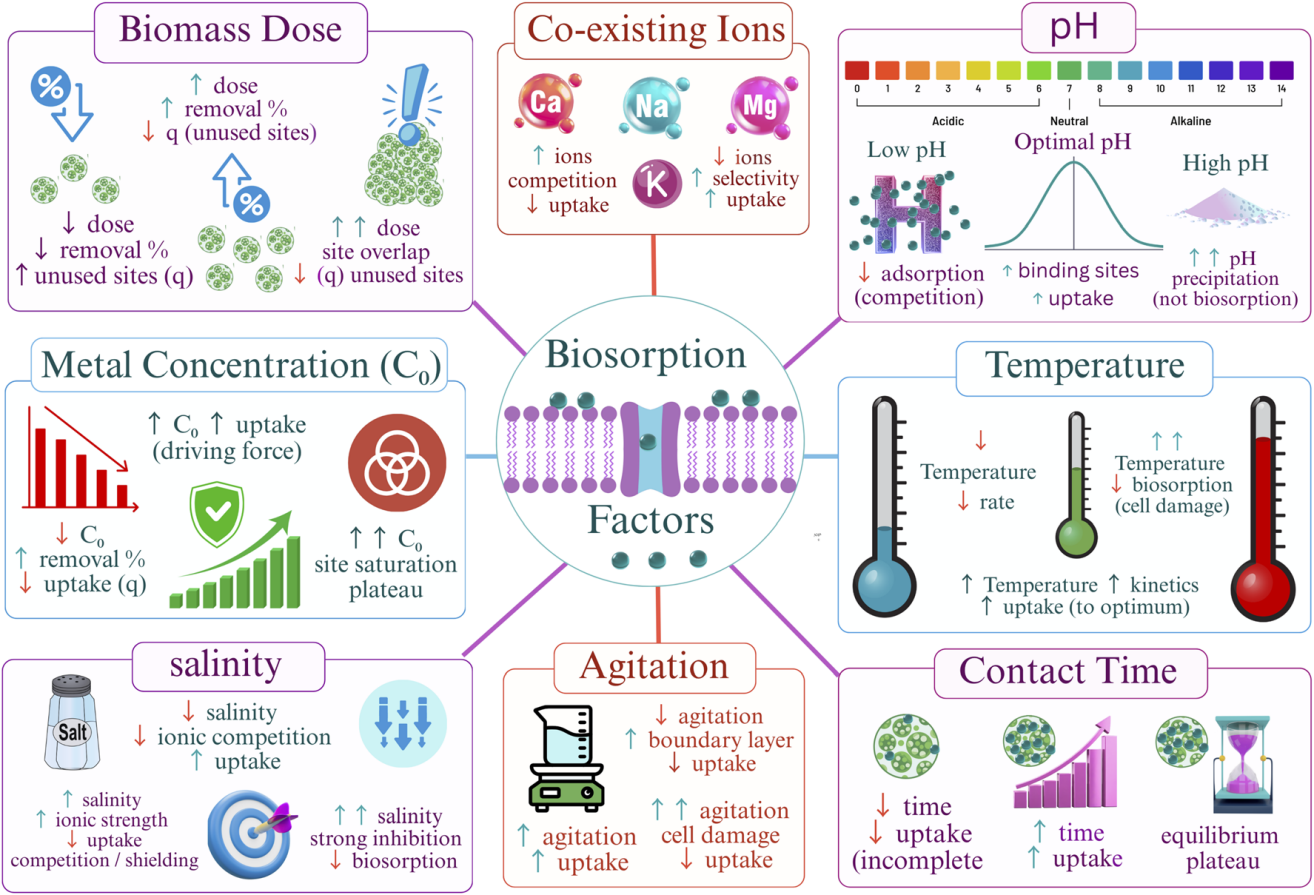
Key physicochemical and operational factors influencing microalgal biosorption of heavy metals, including pH, temperature, contact time, initial metal concentration, salinity, biosorbent dosage, agitation and ionic strength and their collective role in regulating adsorption efficiency and system performance.

Microalgal biosorption efficacy is regulated by a complex interplay of environmental and operational factors (Fig. 2.) that collectively influence metal speciation, surface chemistry, and mass transfer.^62^ These factors also significantly affect metal uptake, tolerance to metal stress, growth, and overall metabolic activity in microalgae.^63^ A thorough understanding of these environmental, operational, and biological factors is essential for optimizing phycoremediation processes and facilitating the scale-up of microalgal technologies for industrial applications.^64^

### 5.1. pH driven speciation and surface charge dynamics

Since pH controls both metal speciation and the ionization state of functional groups on the biomass surface, it is widely recognized as one of the most important factors governing microalgal biosorption.^22^ The ionization state of surface functional groups, including –COOH, –OH, and –NH_2_, as well as the chemical speciation of metals, is directly influenced by pH.^27^ Changes in pH can consequently alter the solubility, reactivity, and bioavailability of metal ions, thereby affecting their interaction with the biosorbent surface.^47^ Under acidic conditions (pH < 4), high concentrations of hydrogen ions (H^+^) or hydronium ions (H_3_O^+^) compete with metal cations for available binding sites on the biosorbent surface.^16^ The resulting high proton concentration promotes the protonation of functional groups, reducing their negative charge and the availability of metal-binding sites, thereby decreasing metal-ion adsorption.^65^

On the other hand, increasing pH promotes deprotonation, which can enhance complexation and electrostatic interactions with positively charged metal ions.^38^ However, at sufficiently high pH values, increasing OH^−^ concentrations can promote the formation and precipitation of metal hydroxides, thereby reducing the fraction of dissolved metal available for interaction with the biomass. Consequently, at high pH values (>7), the apparent removal of heavy metals may partly reflect precipitation rather than biosorption alone, making the contribution of surface binding more difficult to distinguish.^66^ This overlap between adsorption and precipitation complicates the interpretation of experimental results and emphasizes the importance of closely monitoring and reporting pH values. Depending on the metal and microalgal species, the optimal pH range for maximum removal efficiency generally falls between 4 and 7. For instance, *C. sorokiniana* exhibited its highest experimentally determined adsorption capacity at pH 7.^58^ Similarly, the highest removal of Cu(ii), Zn(ii), and Pb(ii) was observed at pH 7 after 60 min of contact time and at an initial metal concentration of 0.5 mg L^−1^; 12.5 mg of biomass removed more than 80% of Zn(ii) and Pb(ii).^16^ The highest adsorption capacities were reported at pH 7.0, reaching 37.1 mg g^−1^ for Cr(vi), 25.98 mg g^−1^ for Cu(ii), 25.06 mg g^−1^ for Pb(ii), and 24.62 mg g^−1^ for Cd(ii).^60^ In contrast, Pb(ii) biosorption exhibited an optimum pH of 5.0, whereas Cd(ii) biosorption showed an optimum pH of 4.0.^67^ Immobilized *Desmodesmus* sp. exhibited maximum Fe(ii) removal efficiency at pH 6.^68^ The marine microalga *D. salina* achieved its highest lead removal (4.5 mg L^−1^) at pH 7–8 and its lowest removal (1.1 mg L^−1^) at pH 12.^69^ These findings demonstrate that pH optimization cannot be generalized across microalgal biosorption systems or defined by a single universal optimum. The effective pH is determined by the combined effects of metal speciation, surface functional-group chemistry, biomass configuration, and the potential contribution of precipitation. Accordingly, pH should be treated not merely as an operational parameter to be optimized for maximum removal, but as a mechanistic variable that determines the relative contributions of surface binding, electrostatic attraction, complexation, and precipitation to the observed removal performance.

### 5.2. Temperature effects on reaction kinetics and thermodynamics

Temperature affects diffusion rates, reaction kinetics, and thermodynamic behavior, all of which influence biosorption. Increasing temperature may initially enhance adsorption by inducing changes in biomass structure, promoting metal-ion diffusion, activating the biosorbent surface, and exposing previously inaccessible binding sites.^70^ However, the effect of temperature depends on the nature of the biosorption process and is therefore highly system-dependent. In endothermic systems, adsorption capacity generally increases with increasing temperature, whereas exothermic systems exhibit the opposite trend.^34^ Nevertheless, at temperatures above 35 °C, adverse effects on biomass and cellular activity may decrease biosorption efficiency.^55^ According to multiple studies, the optimum temperature varies depending on the microalgal species and target metal, but commonly falls between 20 and 35°.^25^ For example, 35 °C was identified as the optimum temperature for the effective treatment of vanadium(iii)-contaminated water using microalgae.^71^ Similarly, another study reported 35 °C as the optimum temperature for the treatment of copper, cadmium, nickel-contaminated water.^72^

*Scenedesmus obliquus* exhibited the highest chromium biosorption performance in both suspended and biofilm growth systems, outperforming *Chlorella vulgaris* at an acidic pH of 5. However, the relative performance of the two species varied with temperature, with *S. obliquus* achieving maximum chromium removal at 26.5 °C in the biofilm system and 27.5 °C in suspension.^18^ These findings demonstrate species-specific responses to environmental conditions, with *C. vulgaris* showing greater performance under acidic conditions, whereas *S. obliquus* performed better at higher temperatures.^18^ Under optimal conditions of an adsorbent dose of 1 g L^−1^, an initial Pb(ii) concentration of 25 mg L^−1^, pH 6, a temperature of 25 °C, and a contact time of 120 min, *Haematococcus pluvialis* achieved a Pb(ii) removal efficiency of 98.69%.^73^ Similarly, under optimal conditions of pH 6.34, a temperature of 27.67 °C, and a biomass dose of 1.5 g L^−1^, *Chlorella kessleri* achieved a maximum Pb(ii) removal efficiency of 99.537%.^74^ Overall, the effect of temperature on microalgal biosorption is not consistent across different species, metals, or biomass configurations. The differences observed between microalgal strains, as well as between suspended and biofilm systems, indicate that the optimum temperature is closely related to the characteristics of the specific biosorption system. Therefore, temperature should be optimized together with the target metal, microalgal species, and biomass configuration rather than treated as an independent operating parameter. In practical applications, the temperature that provides the highest removal efficiency should also be evaluated in terms of biomass stability and energy requirements, particularly during scale-up.

### 5.3. Metal loading and driving force limitations

The initial concentration of metal ions determines the driving force for mass transfer between the liquid phase and algal biomass.^75^ At low metal concentrations, removal efficiency may remain high because the number of available binding sites is relatively large compared with the number of metal ions present.^76^ As the metal concentration increases, the greater concentration gradient enhances the transfer of metal ions toward the biomass surface and increases their interaction with available binding sites, often resulting in a higher adsorption capacity.^77^ On the other hand, as binding sites become progressively saturated, the system approaches equilibrium, and further increases in metal concentration may no longer result in proportional increases in adsorption.^20^ This trade-off highlights the importance of distinguishing between adsorption capacity and removal efficiency when evaluating biosorption performance. Previous research showed that *Chlorella coloniales* was effective in removing Cr(iii), Co(ii), and Fe(ii) at low heavy-metal concentrations; however, its bioaccumulation efficiency decreased at higher metal concentrations.^78^*Coelastrella* sp. exhibited high uptake efficiency for Pb(ii) and Cd(ii), with Pb(ii) removal rates of 86.8%, 86%, 82.85%, and 78% and Cd(ii) removal rates of 84%, 80.66%, 77.14%, and 76.94% under different initial metal concentrations.^79^ Understanding this concentration-dependent behavior and the associated saturation threshold is essential for designing scalable wastewater treatment systems. A recent study demonstrated that the initial concentration of Hg(ii), ranging from 500 to 2000 µg L^−1^, significantly affected the biosorption performance of green microalgae (*Coelastrella* sp.) in an airlift bioreactor, with the highest removal observed at 2000 µg L^−1^.^80^ This finding highlights the importance of optimizing the initial contaminant concentration to improve metal removal performance. *Scenedesmus obliquus*, a freshwater green microalga, removed nearly 95% of Cu(ii) at initial concentrations of ≤0.5 mg L^−1^.^81^ These results show that metal concentration can influence biosorption in different ways depending on the system. Increasing the concentration may initially enhance metal uptake by increasing the driving force for mass transfer, but excessive loading can eventually lead to saturation of available binding sites and reduced removal efficiency. The observed differences among microalgal species and metal ions also indicate that concentration effects cannot be separated from the characteristics of the biomass and the target metal. Therefore, treatment systems should be designed according to the expected concentration range of the wastewater, rather than assuming that higher metal concentrations will always result in better biosorption performance.

### 5.4. Biomass loading and active site saturation

The availability of binding sites in the system is directly influenced by the biomass dose.^17^ By providing a greater number of active sites for metal binding, increasing the biomass concentration generally improves overall removal efficiency.^82^ A recent study demonstrated that removal efficiency increased from 15% to 97% as the adsorbent dose increased; however, the equilibrium adsorption capacity for thorium decreased with increasing biomass dose.^83^ Excessively high biomass doses may promote binding-site aggregation or shielding, thereby reducing the effective surface area.^84^ Furthermore, high biomass concentrations may impose mass-transfer limitations because of increased viscosity and reduced mixing efficiency. Consequently, an optimal biomass dose requires a balance between the availability of active binding sites and efficient mass transfer.^85^

The removal efficiency of Cr(vi) was evaluated at inoculum concentrations of 0.2, 0.4, 0.6, and 0.8 g L^−1^ under autotrophic and heterotrophic conditions. The highest removal efficiencies, 98.9% and 99.9%, respectively, were achieved at an inoculum concentration of 0.8 g L^−1^. Similarly, the adsorption capacities of *Scenedesmus* sp. for Pb(ii) and Cd(ii) initially increased with increasing biomass dose but decreased at higher doses because of biomass aggregation and reduced availability of active binding sites.^86^ Optimizing metal uptake while maintaining overall process efficiency requires the determination of an appropriate biomass dose. The practical application of high biomass doses must also consider the associated economic burdens, as increasing biomass requirements raise the costs of cultivation, harvesting, dewatering, and, where applicable, immobilization. Importantly, higher biomass doses do not necessarily result in proportionally improved performance.^87^ Once adsorption sites become saturated or diffusion limitations arise because of biomass aggregation, further increases in biomass dose may provide only marginal benefits. This saturation effect highlights the importance of strategic dosage optimization.^88^ Optimization strategies should therefore seek to achieve the best balance between removal efficiency and operational cost, thereby supporting the long-term industrial applicability of microalgal biosorption.^89^ Ultimately, biomass dosage should be determined not solely on the basis of maximum removal, but according to cost-efficiency criteria that support scalable and economically viable wastewater treatment. These findings show that increasing the biomass dose does not necessarily lead to a proportional improvement in biosorption performance. Although higher biomass concentrations generally increase the number of available binding sites and may improve removal efficiency, excessive biomass can promote aggregation and mass-transfer limitations while increasing operational costs. Therefore, the optimum biomass dose should be determined by balancing removal efficiency, effective mass transfer, and the economic requirements of biomass production and handling.

### 5.5. Time-dependent uptake and diffusion constraints

In phycoremediation systems, contact time is a crucial factor that controls the effectiveness and kinetics of pollutant removal. It impacts the balance between adsorption, uptake, and desorption processes by determining how long pollutants interact with algal biomass.^90^ Biosorption often occurs in two stages: a quick initial phase brought on by surface binding and a delayed phase associated with diffusion into inner structures or difficult-to-reach areas.^91^ Longer contact times generally result in higher removal efficiency up until a saturation point, after which there is no noticeable increase because of the depletion of active binding sites or equilibrium attainment.^92^ For instance, *Scenedesmus* sp. achieved fast biosorption of Pb(ii) and Cd(ii) within the first 20 minutes, reaching equilibrium at 90 and 60 minutes respectively, after which the removal rate slowed, highlighting the importance of optimizing contact time and the necessity of optimizing contact time for optimal efficiency.^86^

Furthermore, algal species, biomass form (free or immobilized), metal concentration and environmental conditions all influence the required equilibrium time.^93^ Over time, diffusion restrictions often become more significant, particularly in immobilized or aggregated systems where further uptake may be limited by internal mass transfer resistance. *Scenedesmus obliquus* exhibited the maximum chromium absorption under standardized conditions of pH 7, 105 minutes of contact time, 25 °C, 3500 lux of light intensity, and a density of 60.^18^ In addition, microalgae in both suspension and biofilm systems significantly enhanced their uptake of chromium when the contact time was increased from 60 to 115 minutes.^18^

In continuous treatment systems, contact time must be optimized to balance removal efficiency with process throughput and economic feasibility.^94^ While excessively long contact times might not be feasible for large-scale applications, short contact times could result in incomplete removal. Designing effective, economical treatment systems and guaranteeing high throughput in actual wastewater conditions require an understanding of the temporal dynamics of metal uptake by microalgae. These findings show that contact time strongly influences metal uptake, particularly during the initial phase of rapid surface binding. However, extending the treatment time beyond the point at which equilibrium is approached may provide limited additional removal, especially when internal diffusion becomes a limiting factor. Therefore, contact time should be optimized according to the specific metal–biomass system and treatment configuration, while considering the need to balance removal performance with reactor throughput and process efficiency.

### 5.6. Effect of ionic salinity on microalgal biosorption of heavy metals

Salinity exerts both physiological and physicochemical effects on the biosorption of heavy metals by microalgae, making it an important environmental factor. Moderate salinity levels may enhance ATP production and photosynthetic efficiency in certain species, such as *Chlorella* sp., potentially improving biosorption performance.^21^ However, high salinity generally reduces heavy-metal removal efficiency.^95^ The resulting increase in ionic strength compresses the electrical double layer surrounding the algal cell surface or immobilization matrix, thereby weakening electrostatic interactions between negatively charged functional groups on the biomass and metal cations such as Pb(ii) and Cd(ii).^96^ Furthermore, high concentrations of competing ions may directly compete for available binding sites, particularly Na(i).^97^ Species-specific responses to salinity have been reported in numerous studies. For example, increasing salinity decreased the removal efficiencies of Pb(ii) and Cd(ii) in three marine microalgae, namely *Nitzschia* sp., *Chaetoceros* sp., and *Tetraselmis* sp.^98^*Tetraselmis* sp. exhibited superior Cd(ii) removal, whereas Pb(ii) uptake was relatively comparable among the three species, with *Chaetoceros* sp. maintaining the most stable Pb(ii) removal performance.^98^ These findings indicate that, despite species-specific physiological adaptations, each microalga metal combination may have an optimal salinity range beyond which biosorption efficiency declines.^99^ High salinity may also negatively affect chromium removal by *Dunaliella salina*, as the increased ionic strength compresses the electrical double layer surrounding the algal cell surface, thereby weakening electrostatic interactions between chromium species and negatively charged functional groups.^100^ Furthermore, competing ions in saline environments may further inhibit chromium uptake and adsorption efficiency. Overall, salinity influences microalgal biosorption through both physiological responses and physicochemical changes at the biomass–solution interface. Increasing salinity generally enhances ionic competition and electrostatic shielding, although the response remains species- and metal-dependent, highlighting the need to optimize salinity for each microalgal biosorption system.

### 5.7. Ionic strength and competitive binding effects

In actual wastewater systems, heavy metals commonly coexist with other metal ions and dissolved salts.^101^ Functional groups such as carboxyl, hydroxyl, phosphate, and amino groups, which are abundant on the surface of microalgal cells, provide binding sites for metal ions.^30^ These coexisting ions, often referred to as competing ions, may compete for the same binding sites on the algal cell wall and thereby influence metal uptake.^102^ Accordingly, biosorption performance is strongly affected by the presence of competing ions and variations in ionic strength. The outcome of these competitive interactions depends on factors including ionic charge, ionic radius, and affinity for specific functional groups.^103^ Selective adsorption in multimetal systems reflects competition for shared binding sites, while engineered adsorbents can enhance target-metal selectivity through tailored chemical functionalities and coordination environments.^104,105^ The presence of essential divalent cations can also strongly influence metal biosorption by microalgae. For example, a study on *Scenedesmus quadricauda* showed that increasing calcium concentrations decreased heavy-metal uptake, with the magnitude of the effect following the order Cd(ii) < Mn(ii) < Pb(ii), particularly for the absorbed fraction.^106^ This finding suggests that these metals compete with Ca(ii), and that biosorption efficiency may also be influenced by the Ca/Na ratio. Depending on the metal and microalgal species, the adsorption capacity of algal biomass followed the order Cu(ii) > Co(ii) > Cd(ii). Furthermore, *Spirulina platensis* exhibited greater selectivity for Cd(ii) and Co(ii) than *Scenedesmus obliquus* and *Chlorella vulgaris*, whereas *Chlorella vulgaris* showed greater selectivity for Cu(ii) than the other two species.^107^ A recent study reported that *Scenedesmus* sp. cultivated petroleum effluent achieved removal efficiencies ranging from 35% to 96% for Cr(vi), Cd(ii), and Cu(ii), with particularly high chromium removal, highlighting its potential for the phycoremediation of industrial petroleum effluents.^108^ Similarly, *Scenedesmus* sp. effectively removed several heavy metals from landfill leachate in batch, continuous, and membrane bioreactor (MBR) systems, including Pb(ii), Co(ii), Cr(iii), Ni(ii), and Zn(ii).^109^ Zn(ii) exhibited the lowest removal, whereas Ni(ii) showed the highest removal, with concentrations of 0.083 mg L^−1^ in the MBR system and 0.068 mg L^−1^ in the batch system, respectively. These findings highlight the system-dependent nature of biosorption performance and the metal-specific behavior of microalgal systems, which may be influenced by surface interactions and metal speciation.

In some situations, synergistic effects occur, in which the presence of one metal can enhance the uptake of another by altering the cell wall or triggering specialized transport proteins.^50^ A clear trend was noted where nanoscale zero-valent iron promoted microalgal secretion of extracellular polymeric substances, increasing the number of binding sites, organic ligands, and functional groups on the microalgal surfaces and, as a result, enhancing microalgal settling and heavy metal binding.^110^ Furthermore, genetically modified microalgae with greater selectivity for target metals were developed to reduce the effects of competing ions.^111^ Heavy metals and other toxic chemicals may inhibit microalgal growth and photosynthetic efficiency, thereby reducing treatment performance.^112^ To enhance microalgae-mediated pollutant removal in wastewater treatment systems, key operating parameters must be carefully optimized. Increased ionic strength can also shield surface charges and thereby weaken electrostatic attraction between metal ions and the biosorbent surface.^113^ This effect may reduce adsorption efficiency, particularly in systems where electrostatic interactions are the predominant binding mechanism.^114^ Biosorption performance may also be overestimated when assessed in laboratory-scale single-metal systems in the absence of competing ions.^115^ Reliable performance evaluation and prediction for real-world wastewater applications therefore depend on accurately accounting for ion competition and appropriately selecting microalgal species or immobilization strategies.^116^ Therefore, biosorption efficacy in actual wastewater conditions requires an understanding of these shielding and competing effects. Accordingly, understanding these electrostatic shielding and competitive effects is essential for evaluating biosorption performance under realistic wastewater conditions. Collectively, these findings demonstrate that biosorption performance in multicomponent wastewater cannot be reliably predicted from single-metal experiments alone. The presence of competing ions, variations in ionic strength, and metal-specific differences in surface affinity and speciation can substantially alter both the extent and selectivity of metal removal. Although competition may sometimes be offset by synergistic responses, such as enhanced EPS production, these effects remain highly dependent on the microalgal species and wastewater matrix. Therefore, realistic assessment of microalgal biosorption requires the explicit consideration of ionic strength, competing ions, metal speciation, and biomass configuration rather than relying solely on performance data obtained under simplified laboratory conditions.

### 5.8. Effect of agitation intensity on microalgal heavy metal removal

Agitation is crucial for enhancing the heavy-metal removal capacity of microalgal systems by improving mass transfer and reducing external diffusion limitations.^117^ By decreasing the thickness of the boundary layer at the solid–liquid interface, increased mixing intensity promotes solute diffusion from the bulk solution to active surface sites. Because external mass transfer often controls the initial uptake stage in liquid-phase adsorption systems, the resulting reduction in film-diffusion resistance can increase the overall adsorption rate.^118^ Adequate agitation also prevents biomass settling and promotes contact between biosorbent particles and metal ions, thereby improving system homogeneity and increasing the effective surface area available for adsorption.^119^ However, excessive agitation can generate hydrodynamic shear stress that damages microbial or algal cell integrity and reduces biosorption efficiency in biological systems.^118^ Accordingly, the decline in removal efficiency observed at agitation speeds above the optimum is more likely to result from excessive hydrodynamic shear stress, which can disrupt biomass structure and weaken the stability of metal–biosorbent interactions.^120^ Therefore, optimizing agitation intensity is essential in microalgal heavy-metal removal systems to balance enhanced mass transfer against the structural stability of the biosorbent. Overall, agitation intensity has a dual effect on microalgal biosorption: moderate mixing enhances mass transfer and metal-biomass contact, whereas excessive agitation may damage biomass structure and reduce removal efficiency. Therefore, agitation should be optimized according to the specific biomass configuration to achieve an appropriate balance between mass-transfer enhancement and biosorbent stability.

### 5.9. Nonlinear and interactive effects of operational parameters

In actual biosorption systems, operational parameters interact in complex and nonlinear ways rather than acting independently.^121^ For example, the effect of pH on adsorption capacity may depend on temperature, ionic strength, and metal concentration.^122^ Biomass dose and contact time can also influence diffusion limitations and surface accessibility.^123^ These interactions often produce antagonistic or synergistic effects that are not captured by single-factor analyses.^124^ Consequently, understanding biosorption behavior requires a system-level perspective that considers the combined effects of multiple factors. This complexity highlights the importance of integrated modeling approaches and multivariate optimization for accurately predicting and improving biosorption performance.

Microalgal biosorption performance is determined by the combined effects of environmental and operational conditions rather than by any single parameter. pH, temperature, metal concentration, biomass dose, contact time, salinity, ionic strength, and agitation can each alter metal speciation, surface chemistry, mass transfer, or the availability of binding sites, while their interactions may further modify the final removal outcome. The conditions that maximize removal efficiency do not necessarily maximize adsorption capacity or process feasibility. Therefore, optimizing microalgal biosorption requires a system-specific approach that considers the target metal, biomass characteristics, wastewater composition, and interactions among operating variables rather than relying on isolated single-factor optimization. These findings indicate that the effects of individual operating parameters cannot be interpreted independently, as their interactions may substantially influence biosorption performance. Therefore, the optimum conditions identified for one system may not be directly applicable to another, highlighting the need for integrated, system-specific optimization rather than isolated single-factor analysis.

## 6. Process modelling and predictive descriptions of biosorption

### 6.1. Equilibrium modelling

**Fig. 3:**
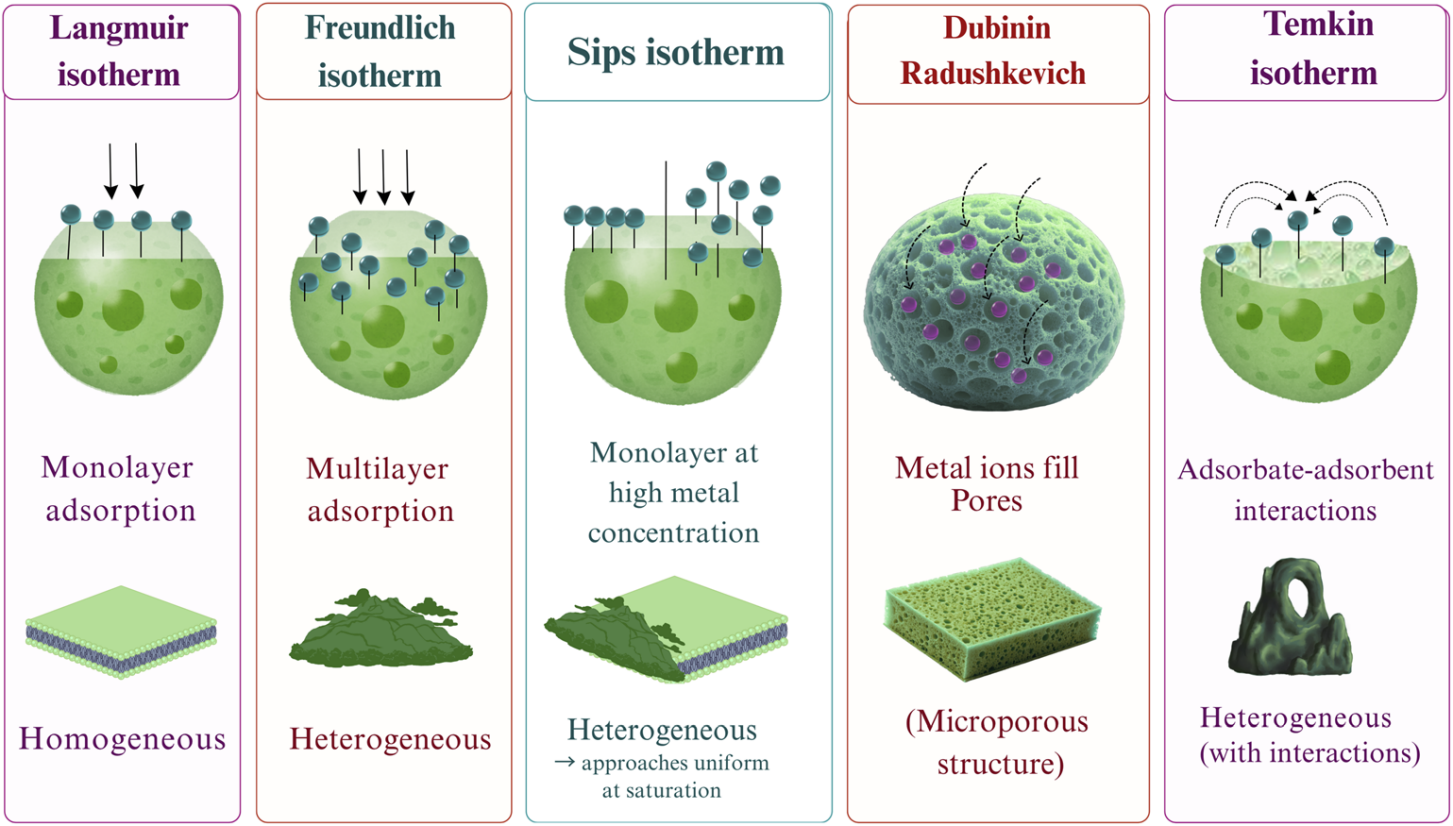
Overview of commonly applied adsorption isotherm models used in biosorption studies, including Langmuir, Freundlich, Temkin, and Dubinin Radushkevich models, describing equilibrium relationships between heavy metal ions and microalgal biomass.

To describe the distribution of metal ions between the aqueous phase and microalgal biomass at equilibrium, equilibrium modeling is essential (Table 1.) (Fig. 3). The Langmuir isotherm is one of the most widely used models and assumes monolayer adsorption onto a homogeneous surface with a finite number of identical binding sites.^125^ This model provides a simplified representation of biosorption systems and is particularly useful for estimating the maximum adsorption capacity.^126^ A recent study reported, based on Langmuir isotherm modeling, that the adsorption process was predominantly associated with monolayer chemisorption.^127^ For example, the biosorption of lead and cadmium, with monolayer capacities of 102 and 128 mg g^−1^, respectively, was best described by the Langmuir model.^86^ However, the assumption of surface homogeneity does not fully reflect the complex and heterogeneous structure of microalgal cell walls.^128^

**Table 1:** Adsorption isotherm models and their mathematical expressions

Models	Langmuir	Freundlich	Sips	Dubinin–Radushkevich	Temkin
Governing equation	*q*_e_ = (*K*_L_ × *C*_e_)/(1 + *K*_L_ × *C*_e_)	*q*_e_ = *K*_F_ × *C*_e_^(1/*n*)	*q*_e_ = (*q*_max_ × *K*_s_ × *C*_e_^*n*)/(1 + *K*_s_ × *C*_e_^*n*)	*q*_e_ = *q*_m_ × exp(−*β* × *ε*^2^)	*q*_e_ = *B* ln(*A* × *C*_e_)
				*ε* = *R* × *T* × ln(1 + 1/*C*_e_)	*B* = (*R* × *T*)/*b*
				*E* = 1/sqrt(2 × *β*)	
Key parameters (definition)	*q*_e_: adsorbed amount (mg g^−1^), *q*_max_: maximum capacity (mg g^−1^), *K*_L_: affinity constant (L mg^−1^), *C*_e_: equilibrium concentration (mg L^−1^)	*K*_F_: adsorption capacity, 1/*n*: heterogeneity factor	*K*_s_: Sips constant, *n*: heterogeneity index	*q*_m_: capacity, β: energy constant	*A*: binding constant
Key assumption	Monolayer adsorption on homogeneous surface with identical sites and no interaction	Multilayer adsorption on heterogeneous surface	Hybrid: Freundlich at low *C*_e_, Langmuir at high *C*_e_	Pore filling mechanism	Heat of adsorption decreases linearly
Strengths	Simple, mechanistic, estimates maximum capacity	Flexible, strong empirical fit	Combines Langmuir + Freundlich	Identifies adsorption energy	Accounts for interactions
Limitations	Unrealistic for heterogeneous biomass	No saturation limit	More complex fitting	Weak at high concentration	Limited applicability
Applicability	Idealized lab systems	Real wastewater systems	Real biosorption systems	Microporous systems	Moderate interaction systems
Keywords	Monolayer, saturation	Heterogeneous, empirical	Hybrid model	Energy-based	Interaction
References	129 and 130	131	132 and 133	130 and 131	129 and 130

However, the Freundlich isotherm is more appropriate for systems with a variety of functional groups and non-uniform binding affinities since it accounts for heterogeneous surface energies and multilayer adsorption.^134^ The Freundlich model often provides a better fit to experimental biosorption data while being empirical.^135^ Dried *Chlorella* sp. effectively removed Cd(ii) according to Freundlich behavior (*R*^2^ = 0.9829), exhibiting heterogeneous multilayer adsorption.^58^ However, both models are mostly descriptive and lack mechanistic depth, which limits their capacity to faithfully depict actual biosorption systems in a variety of environmental settings. By combining principles of the Freundlich and Langmuir isotherms, the Sips isotherm model is a hybrid equation that may be used to predict monolayer adsorption capacity at high concentrations and describe adsorption on heterogeneous surfaces.^132^ The Sips model is frequently used in the context of microalgal biosorption for systems that show both saturation behavior and heterogeneous binding sites, especially in environments like wastewater matrices. The mechanistic complexity of biosorption processes dictated by biological variability and surface functional variety is not fully captured by this empirical representation, despite its flexibility and better fitting performance when compared to classical models.^136^ The Sips isotherm model offered the best match for living microalgal systems, allowing the estimate of maximum adsorption capacity (*q*_max_), according to equilibrium modelling of Cd(ii) biosorption by Nordic microalgae.^137^

The Dubinin–Radushkevich (D–R) isotherm model is frequently employed in biosorption studies to elucidate the characteristics of metal–biosorbent interactions and the underlying adsorption mechanism.^138^ Unlike conventional isotherm models, the D–R model does not assume a homogeneous surface or a constant adsorption potential, making it suitable for heterogeneous biosorbents such as microalgal biomass.^139^ One of the key advantages of this model is its ability to estimate the mean free energy of adsorption, which can provide insights into whether the adsorption process is predominantly physical or chemical in nature.^139^ The D–R model has been applied to microalgal systems to assess the contribution of surface adsorption and pore-filling mechanisms to heavy-metal removal.^140^ Despite its utility, the model remains semi-empirical and may not accurately capture the complexity of biosorption processes governed by diverse functional groups and biological heterogeneity on algal cell surfaces.^58^ The Dubinin–Radushkevich isotherm, a two-parameter adsorption model, showed good agreement with equilibrium data and successfully characterized As(iii) biosorption by *Chlorella vulgaris*.^141^

The Temkin isotherm model assumes that the heat of adsorption decreases linearly with increasing surface coverage and accounts for adsorbate–adsorbent interactions.^142^ This approach provides useful thermodynamic insights into biosorption systems by considering indirect interactions between adsorbed metal ions and the biosorbent surface.^143^ The Temkin model is particularly applicable to microalgal biosorption systems in which lateral interactions between adsorbed species influence overall uptake behavior and binding energies are non-uniform.^144^ In a recent study, *Dunaliella* sp. was immobilized in calcium alginate beads and evaluated for Cu(ii) removal in batch and fixed-bed column systems. The equilibrium data were best fitted by the Freundlich and Temkin models, indicating heterogeneous adsorption and interactions between the adsorbate and biosorbent.^129^ However, the simplification of complex biological surface heterogeneity and its semi-empirical nature continue to limit the mechanistic interpretation provided by the Temkin model.

Overall, the reviewed studies indicate that no single isotherm model can universally describe microalgal biosorption. The suitability of each model depends on the characteristics of the biomass, the target metal, and the experimental conditions. Langmuir modeling is useful for estimating maximum adsorption capacity and describing saturation behavior, whereas Freundlich, Sips, D–R, and Temkin models may provide better representations of heterogeneous surfaces, variable binding energies, and adsorbate–biosorbent interactions. However, the statistical fit of an isotherm model should not be interpreted as definitive evidence of a specific adsorption mechanism. Therefore, the selection and interpretation of equilibrium models should combine goodness-of-fit with physicochemical characterization and mechanistic evidence, particularly when extrapolating laboratory results to complex wastewater systems.

### 6.2. Kinetic descriptions and transport-limited behavior

The rate and controlling stages of the biosorption process are revealed by kinetic modelling.^145^ Kinetic modeling provides insights into the rate and controlling stages of the biosorption process.^50^ Metal uptake kinetics are commonly described using pseudo-first-order and pseudo-second-order models, with the pseudo-second-order model often showing a better fit to experimental data (Table 2). Although such results should be interpreted cautiously, a better fit to the pseudo-second-order model is often considered indicative of chemisorption.^146^ For example, the biosorption of Cr(vi) by biomass derived from the microalgal isolate *Asterarcys* sp. and a mixed algal community was adequately described by the pseudo-second-order model, suggesting that chemisorption may have been a major rate-controlling mechanism.^147^ In contrast, kinetic studies of *Chlorella* sp. biomass for heavy-metal removal showed a better fit to the pseudo-first-order model.^16^

**Table 2:** Comparative overview of adsorption kinetic and thermodynamic models in biosorption systems

Model type	Kinetic	Kinetic	Kinetic	Thermodynamic
Model	Pseudo-first order	Pseudo-second order	Intra-particle diffusion	Van't Hoff
Governing equation	d*q*_*t*_/d*t* = *k*_1_ × (*q*_e_ − *q*_*t*_)	d*q*_*t*_/d*t* = *k*_2_ × (*q*_e_ − *q*_*t*_)^2^	*q*_*t*_ = *k*_id_ ×*t*^(1/2)^ + *C*	ln *K* = −(Δ*H*°/(*R* × *T*)) + (Δ*S*°/*R*), Δ*G*° = –*R* × *T* ln *K*
Key parameters (definition)	*q*_*t*_: Adsorbed amount at time *t*, *k*_1_: rate constant (min^−1^)	*k*_2_: rate constant (g mg^−1^ min^−1^)	*k*_id_: diffusion constant, *C*: boundary layer thickness	Δ*G*°: spontaneity, Δ*H*°: enthalpy, Δ*S*°: entropy, *K*: equilibrium constant
Key assumption	Rate proportional to unoccupied sites	Chemisorption-controlled process	Diffusion inside particles is rate-limiting	Adsorption governed by energy changes
Strengths	Simple and widely used	Excellent fit for most systems	Identifies diffusion control	Evaluates feasibility and nature
Limitations	Limited to initial stage	Not mechanistic proof	Oversimplified model	Needs multi-temperature data
Applicability	Early adsorption phase	Most biosorption systems	Porous and immobilized systems	All adsorption systems
Keywords	Kinetics, diffusion	Chemisorption	Diffusion, transport	Thermodynamics, energetics
References	148 and 146	149 and 150	150 and 151	152–154

Beyond empirical kinetic models, biosorption rates are strongly influenced by mass-transfer limitations. Metal uptake can be significantly affected by processes such as film diffusion and intraparticle diffusion, particularly in systems containing aggregated or immobilized biomass.^146^ Under certain conditions, mass-transfer resistance may become the primary limiting factor rather than the surface reaction kinetics. Therefore, a comprehensive kinetic analysis should consider both diffusion-controlled and reaction-controlled processes to accurately evaluate system behavior.^155^ For example, Pb(ii) adsorption by sodium alginate-immobilized *Chlorella sorokiniana* FK–montmorillonite gel beads followed a pseudo-second-order kinetic model and a Langmuir isotherm, while intraparticle diffusion was identified as the rate-limiting step governing the adsorption process.^114^ Similarly, under optimized conditions, dead cells of *Microcystis aeruginosa* removed more than 90% of Cd(ii), Pb(ii), and Zn(ii), with the adsorption process following the Langmuir isotherm and pseudo-second-order kinetics, suggesting a predominant chemisorption mechanism.^66^ These findings indicate that the kinetic behavior of microalgal biosorption is highly system-dependent and cannot be reliably interpreted from the best-fitting kinetic model alone. Although pseudo-first-order and pseudo-second-order models can provide useful descriptions of uptake behavior, diffusion limitations may also control the overall rate, particularly in aggregated or immobilized biomass. Therefore, reliable kinetic interpretation requires consideration of both model fitting and mass-transfer processes rather than assigning a specific adsorption mechanism solely on the basis of the kinetic model that provides the best statistical fit.

### 6.3. Thermodynamic interpretation of biosorption feasibility

Thermodynamic analysis is a standard approach for evaluating the feasibility and energetic characteristics of biosorption processes.^143^ Thermodynamic parameters, including the Gibbs free energy change (Δ*G*°), enthalpy change (Δ*H*°), and entropy change (Δ*S*°) are commonly determined to evaluate the spontaneity and energy changes associated with metal uptake (Table 2). For example, thermodynamic analysis indicated that Cu(ii) biosorption was a spontaneous and endothermic process accompanied by a modest increase in interfacial disorder, as reflected by negative Δ*G*°, positive Δ*H*°, and positive Δ*S*° values, possibly due to ion–exchange interactions.^143^ In contrast, negative Δ*G*° and Δ*H*° values indicated that Cu(ii) biosorption using a *Spirulina platensis*-based biosorbent was spontaneous and exothermic.^156^ Changes in the degree of disorder at the solid–solution interface during biosorption are reflected by variations in entropy (Δ*S*°).^157^ Positive Δ*S*° values may indicate an increase in system disorder, which is often associated with the displacement of water molecules during metal binding. For example, a positive Δ*S*° value of 0.068 kJ mol^−1^ indicated an increase in disorder at the solid–liquid interface during biosorption.^158^ These findings indicate that thermodynamic parameters can provide useful information about the spontaneity and energy changes associated with microalgal biosorption, but their interpretation remains highly system-dependent. Differences in the signs and magnitudes of Δ*G*°, Δ*H*°, and Δ*S*° among studies reflect variations in the target metal, biosorbent characteristics, and operating conditions. Therefore, thermodynamic parameters should be interpreted alongside kinetic, equilibrium, and mechanistic evidence rather than used independently to define the biosorption mechanism.

However, these thermodynamic interpretations are often based on oversimplified assumptions and equilibrium conditions that may not accurately capture the dynamic nature of real biosorption systems.^159^ Therefore, thermodynamic results should be interpreted with caution when extrapolating them to real-world conditions.

### 6.4. Linking models with mechanistic reality and critical limitations of conventional modelling frameworks

In adsorption research, bridging the gap between practical biosorption systems and their underlying physicochemical mechanisms remains a significant challenge.^160^ Although kinetic and isotherm models are widely used to fit experimental data, they often fail to accurately capture the complexity arising from diverse binding sites, heterogeneous functional groups, and changing environmental conditions.^161^ The substantial structural and compositional heterogeneity of biomass further influences adsorption behavior in microalgal systems, as surface chemistry and binding affinity vary according to species, cultivation conditions, and physiological state.^70^ Consequently, a single simplified model may not adequately represent the actual biosorption mechanisms occurring in such complex systems.

Integrated, mechanistically informed modeling approaches that account for surface heterogeneity, mass-transfer effects, and system-level variability are therefore required to improve predictive capability.^162^ Traditional biosorption modeling frameworks have several inherent limitations, as many studies rely primarily on curve-fitting approaches without rigorously evaluating whether the selected models accurately reflect the underlying mechanisms. Moreover, these models often assume idealized conditions, including homogeneous surfaces, equilibrium states, and the absence of competitive interactions, which rarely occur in real wastewater systems containing multiple pollutants and complex matrices. Another critical limitation is the inadequate consideration of scale-dependent factors, particularly hydrodynamics and diffusion limitations, which become increasingly important during process scale-u, Consequently, models developed under controlled laboratory conditions may fail to predict performance at larger scales. Addressing these limitations requires modeling frameworks that integrate mechanistic understanding with surface heterogeneity, mass-transfer behavior, and system-level variability to improve predictive accuracy. Such developments are essential for advancing microalgal biosorption from controlled laboratory studies toward reliable real-world wastewater treatment applications.^55^

Overall, adsorption models should be interpreted as tools for describing specific aspects of biosorption rather than as definitive evidence of the underlying mechanism. Equilibrium, kinetic, and thermodynamic models provide complementary information on capacity, uptake rate, transport limitations, and energetic feasibility, but their parameters are strongly dependent on the experimental conditions and assumptions used. The complex and heterogeneous nature of microalgal biomass further limits the ability of any single model to represent the full biosorption process. Meaningful interpretation therefore requires integrating model fitting with independent mechanistic evidence, including surface characterization, metal speciation, diffusion analysis, and evaluation of competitive interactions. Such an integrated approach is essential for distinguishing genuine mechanistic understanding from statistical agreement between experimental data and an empirical model.

## 7. Biological and material diversity as determinants of biosorption performance

### 7.1. Physiological state of microalgal biomass and biosorption performance

Live microalgae provide a biologically active platform for metal removal through the combined action of metabolism-dependent processes, including membrane transport, intracellular sequestration, and biotransformation, together with passive surface biosorption.^163^ Energy-dependent transport systems, enzymatic activity, and membrane integrity enable complex interactions with metal ions and may provide additional selectivity and removal pathways compared with non-viable biomass under suitable physiological conditions.^55^ Accordingly, metal removal by living microalgae may involve both active intracellular uptake and passive binding to cell-surface functional groups, including carboxyl, hydroxyl, sulfate, and phosphate groups. For example, a comparative study of living microalgae demonstrated species-dependent differences in the removal of copper and cadmium, with *Chlorella vulgaris* achieving up to 90% copper removal and exhibiting faster uptake kinetics and higher binding capacities than *Scenedesmus obliquus*.^164^ In addition to metal removal, living microalgal systems may offer advantages such as selective uptake, simultaneous removal of multiple pollutants, and the potential for biomass valorization.^165^ However, their performance depends on physiological activity and can be adversely affected by environmental stressors, including high metal concentrations, toxic organic compounds, and extreme pH conditions.^166^

In living systems, competition between cellular growth and metal uptake may influence removal performance, particularly under conditions in which metal concentrations are insufficient to drive substantial uptake or cellular resources are preferentially allocated to growth. Long-term operation may also be constrained by cell lysis and the potential re-release of accumulated metals.^77^ By contrast, non-viable microalgal biomass relies primarily on passive surface interactions and is generally less dependent on light, nutrients, and metabolic activity, making it attractive for applications requiring greater operational flexibility.^167^ The absence of metabolic requirements can also improve storage and handling stability and reduce the influence of biological activity on treatment performance.^88^ Moreover, non-viable biomass may represent a cost-effective option for large-scale applications because it does not require specific environmental conditions, such as light, nutrients, or controlled temperature (Table 3). In addition, non-viable biomass may tolerate fluctuations in pH, temperature, and metal loading more effectively than living cells.^168^ For example, under optimized conditions, dead *Microcystis aeruginosa* cells removed more than 90% of Cd(ii), Pb(ii), and Zn(ii), with the experimental data being described by the Langmuir isotherm and pseudo-second-order kinetic models.^66^ However, the interpretation of these models should not, by itself, be considered definitive evidence of a predominant chemisorption mechanism.^169^

**Table 3:** Microalgal biomass types and their biosorption characteristics

Biomass type	Live biomass	Dead biomass	Immobilized biomass	Modified biomass	Biofilm systems
Dominant mechanism	Bioaccumulation + passive biosorption	Passive surface biosorption	Diffusion + surface binding	Enhanced chemical binding	EPS-mediated biosorption + bioaccumulation
Rate-controlling step	Membrane transport + metabolic uptake	Film diffusion + surface adsorption	Intraparticle diffusion + external mass transfer	Surface reaction + adsorption kinetics	Multi-layer diffusion + biofilm mass transfer limitation
Surface chemistry interactions	Ion exchange, intracellular complexation, active transport	Electrostatic attraction, ion exchange, surface complexation	Surface complexation within polymer matrix	Functional group engineering (–COOH, –OH, –NH_2_), redox interactions	EPS functional groups (polysaccharides, proteins, carboxyl groups)
Key advantages	Dual removal pathways, metal transformation capability	Stable, low cost, fast uptake, no metabolic dependence	Easy separation, reusable, mechanically stable	Very high capacity and selectivity	High stability, self-regeneration, tolerant to fluctuations
Limitations	Sensitive to toxicity, requires nutrients/light, slower kinetics	Limited selectivity, no intracellular uptake	Mass transfer resistance, higher preparation cost	Costly modification, potential secondary pollution	Complex control, scale-up challenges
Best application	Low metal concentration, phycoremediation systems	High metal concentration, batch systems	Continuous flow systems, large-scale treatment	Targeted metal removal in mixed systems	Industrial wastewater, dynamic systems
Relative performance	Moderate–High	High	High	Very high	High
Reusability potential	Low–Moderate	High (after desorption cycles)	High	Moderate	High (self-renewing)
Key influencing factors	sensitive to high metal levels, organics, and pH extremes	resistant to metals, pH, temperature; high stability	stable under metals and pH; less sensitive to changes	enhanced binding; reduced pH sensitivity	High tolerance to metals and stress; strong stability
References	170–172	173 and 174	127 and 175	176 and 177	170–172

The choice between living and non-viable microalgal biomass depends on wastewater characteristics and specific treatment objectives. Although living systems may offer higher performance under favorable conditions, practical constraints, including biomass recovery, structural fragility, and loss of metabolic activity, have encouraged the development of alternative biomass configurations.^55^ Microalgal biofilms, in which cells are embedded within structured extracellular polymeric substances (EPS), represent one such configuration and may improve biomass retention, stress tolerance and biosorption performance.^168^ By providing a structured matrix that enhances cell retention and creates additional extracellular binding domains, biofilm growth may improve the stability and overall biosorption capacity of microalgal systems under variable environmental conditions. In addition, chemical modification of microalgal biomass can enhance biosorption by introducing or increasing the accessibility of functional groups such as carboxyl, amino, and hydroxyl moieties.^171^

### 7.2. Engineered and immobilized biosorbent platforms

Immobilized biosorbent systems have been developed to overcome several limitations associated with free microalgal biomass.^178^ Immobilization strategies, including attachment to solid supports and entrapment within polymeric matrices, can improve biomass retention and stability, facilitate solid–liquid separation, and support biomass recovery and reuse in continuous-treatment systems.^179^ By reducing biomass loss and improving resistance to environmental fluctuations, immobilization can enhance the operational stability of microalgal biosorption systems.^180^ However, the immobilization matrix may also introduce additional diffusion resistance and limit the accessibility of metal ions to active binding sites, thereby creating a trade-off between biomass stability and mass-transfer efficiency.^178^ Consequently, the design of immobilized biosorbents requires careful optimization of matrix structure, biomass accessibility, mechanical stability, and mass transfer.^171^ In parallel, surface functionalization and composite formation have been investigated as chemical and materials-engineering strategies to modify the surface properties of microalgal biomass and improve its adsorption capacity, selectivity, and operational performance.^114^ These engineered biosorbent platforms provide promising strategies for improving the effectiveness and scalability of microalgal biosorption. For example, immobilized *Chlorella vulgaris* incorporated into a copolymer matrix achieved more than 95% removal of Cu(ii) and Cd(ii) within 8 h.^181^ In another study, immobilization of *Synechocystis* sp. *PCC 6803* using chitosan improved adsorption performance and stability compared with other carrier materials. Under the reported optimum conditions, the system exhibited substantial removal capacities for Cr(vi), Cu(ii), Pb(ii), and Cd(ii), together with an effective desorption rate of approximately 90% and sustained performance over multiple reuse cycles.^60^

### 7.3. Selection criteria for high-performance biosorbents

**Fig. 4:**
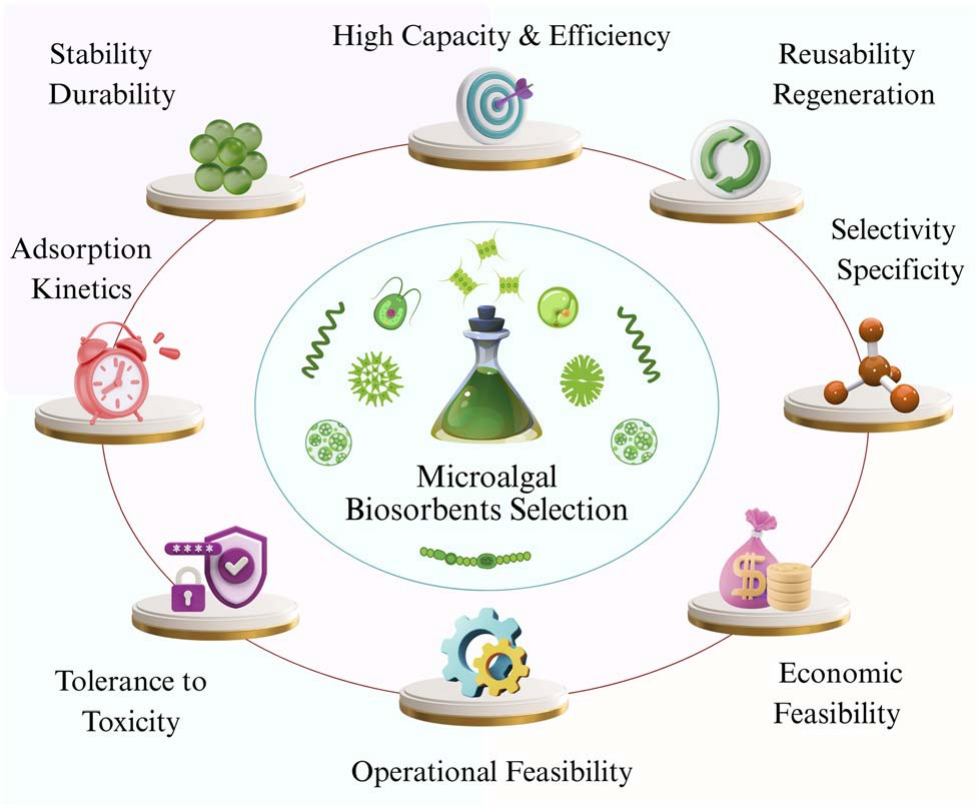
Multi-criteria framework for selecting microalgal biosorbents for heavy-metal removal. Overview of the key criteria influencing biosorbent selection, including adsorption performance, uptake kinetics, selectivity, operational feasibility, physicochemical stability, reusability, and economic considerations.

The selection of an appropriate microalgal biosorbent is a critical determinant of the overall efficiency, cost-effectiveness, and scalability of heavy-metal removal processes. A high-performance biosorbent should ideally combine high metal-binding capacity, rapid and reproducible uptake kinetics, adequate selectivity, mechanical and physicochemical stability, regeneration potential, and acceptable operating costs.^88^ Accordingly, biosorbent selection should consider not only adsorption capacity but also selectivity, biomass availability, production cost, processing requirements, and the feasibility of integration into the intended treatment configuration (Fig. 4).^182^ High functional-group density, favorable surface properties, and structural stability under relevant environmental conditions are additional characteristics that may support efficient and reliable biosorption performance.^38^

High removal efficiency and biosorption capacity remain important selection criteria because the biosorbent must achieve substantial metal removal while providing adequate uptake capacity per unit mass of biomass.^55^ However, capacity alone is insufficient to determine practical suitability. Non-viable biomass may offer advantages under conditions in which metabolic activity is unnecessary, particularly because passive surface binding can be maintained without the light, nutrients, and physiological conditions required by living cells.^22^ Conversely, living biomass and biofilm-based systems may provide additional biological functions and enhanced tolerance under specific environmental conditions through active metabolism and EPS-mediated protection.^183^ The relative advantage of each biomass type therefore depends on the target metal, wastewater composition, treatment configuration, and operational conditions rather than on the existence of a universally superior biomass state. Tolerance to metal toxicity and complex wastewater conditions is another important selection criterion. In addition, practical biosorbents should maintain their removal performance with limited capacity loss over repeated adsorption–desorption cycles. Immobilized and non-viable biomass often offer advantages in recovery and reuse compared with free-living cells, although actual regeneration performance depends on the stability of biomass-metal interactions and the regeneration conditions employed.^82^

For practical applications, efficient solid–liquid separation is essential, particularly when the biosorbent is used in continuous-treatment systems. Immobilized and biofilm-based configurations may facilitate biomass recovery and reduce biomass loss during operation.^184^ Economic feasibility also depends on low-cost biomass production, simple processing, and the potential utilization of waste or residual biomass streams. Chemical modification may further improve metal binding or selectivity in mixed-metal systems by altering the availability and chemical environment of surface functional groups; however, the benefits of modification must be evaluated against additional preparation costs, regeneration requirements, and the potential for secondary contamination.^185^ Rapid and reproducible uptake, together with experimentally validated kinetic behavior, is also relevant to process design. However, the selection of isotherm and kinetic models should be based on mechanistic plausibility and experimental validation rather than statistical fit alone. Therefore, biological properties must be evaluated alongside technical, environmental, and economic considerations when identifying biosorbents suitable for real-world applications.

Overall, the comparison of different microalgal biomass configurations shows that no single format can be considered universally superior for heavy-metal biosorption. The relative performance of live, non-viable, immobilized, modified, and biofilm-based systems depends on the balance among metal-binding capacity, selectivity, mass-transfer accessibility, operational stability, regeneration potential, and the physicochemical complexity of the treated wastewater. Accordingly, biosorbent selection should not be based solely on the highest reported removal efficiency or adsorption capacity obtained under optimized laboratory conditions. Instead, the most appropriate biosorbent should be selected according to the target metal, competing ions, wastewater composition, treatment configuration, biomass recovery requirements, and the feasibility of regeneration and reuse. This comparative perspective shifts biosorbent selection from a capacity-centered approach toward a multidimensional assessment of performance, operational feasibility, and process suitability.

## 8. Critical analysis and research gaps

### 8.1. Complexity of real wastewater systems and engineering constraints

The performance of microalgal biosorption in real wastewater is often substantially more difficult to predict than under simplified laboratory conditions, Table 4. Unlike synthetic solutions containing a single metal ion or a defined mixture of contaminants, industrial effluents contain complex combinations of heavy metals, organic compounds, dissolved salts, suspended solids, and competing ions, together with variable pH, ionic strength, and redox conditions.^186^ These matrix components can compete for available binding sites, modify the surface charge of the biomass, alter metal speciation, or form soluble complexes that reduce the fraction of metal available for interaction with the biosorbent.^58^ Consequently, removal efficiencies and adsorption capacities obtained under simplified laboratory conditions may not accurately represent performance in complex wastewater matrices.

**Table 4:** Laboratory *vs.* real wastewater biosorption performance

Aspect	Synthetic (laboratory) systems	Real wastewater systems	Scientific implication
Composition	Single or binary metal solutions	Multi-contaminant matrix (metals, organics, ions)	Laboratory systems overestimate adsorption performance
Environmental control	Strictly controlled pH, temperature, ionic strength	Highly variable physicochemical conditions	Reduced reproducibility under field conditions
System complexity	Simplified adsorption environment	Competitive adsorption and interference effects	Selectivity becomes a limiting factor
Reproducibility	High experimental consistency	Low due to wastewater heterogeneity	Data variability increases uncertainty
Adsorption performance	Often higher apparent capacity	Generally reduced effective capacity	Performance overestimation in lab studies
Interference effects	Minimal or absent	Significant (competing ions, salts)	Strong impact on surface binding sites
Practical relevance	Limited to mechanistic understanding	High environmental and industrial relevance	Essential for scale-up validation
References	82 and 187	188 and 189	27 and 17

In multi-contaminant environments, heavy metals and organic contaminants may coexist and compete for the same or overlapping binding domains on microalgal biomass.^190^ Such competition can alter the apparent selectivity of the biosorbent and reduce uptake of the target metal, particularly when competing species exhibit greater affinity for specific functional groups or when their charge and chemical speciation favor stronger interactions with the biomass. Conversely, interactions among coexisting contaminants may occasionally produce non-additive effects, including apparent synergistic or antagonistic behavior. These complexities are particularly relevant to industrial effluents from sectors such as mining, electroplating, and textile processing, where wastewater composition, contaminant concentrations, flow rates, and reactor configurations can vary substantially.^191^

Although integration with existing treatment processes and the use of complementary technologies have shown promise, maintaining stable long-term performance remains a major engineering challenge, particularly under continuous-flow conditions. Changes in wastewater composition, biomass condition, hydraulic loading, and contaminant concentration can progressively alter biosorption performance. This exposes a major limitation of much of the current biosorption literature: many studies evaluate performance under static and highly controlled conditions, whereas industrial treatment systems operate under dynamic and chemically heterogeneous conditions. A critical research gap therefore lies in developing experimental and modeling approaches capable of describing multi-component competition, temporal variability, and matrix-dependent biosorption behavior under realistic operating conditions.

### 8.2. Limitations in predictive biosorption modeling

The predictive capacity of current microalgal biosorption models remains limited by a persistent disconnect between the complexity of the underlying biological and physicochemical processes and the simplified assumptions used in many conventional modeling approaches. Microalgal biosorption operates across multiple scales, from metal speciation and interfacial chemistry to biomass structure, mass transfer, and reactor-level performance.^55^ Operational variables such as pH, temperature, contact time, ionic strength, and initial metal concentration influence the system not as isolated parameters, but through their effects on surface reactivity, metal speciation, binding-site accessibility, and transport processes.^128^ At the interfacial level, simultaneous mechanisms including ion exchange, surface complexation, electrostatic attraction, and precipitation may contribute to metal removal.^20,40^

**Fig. 5:**
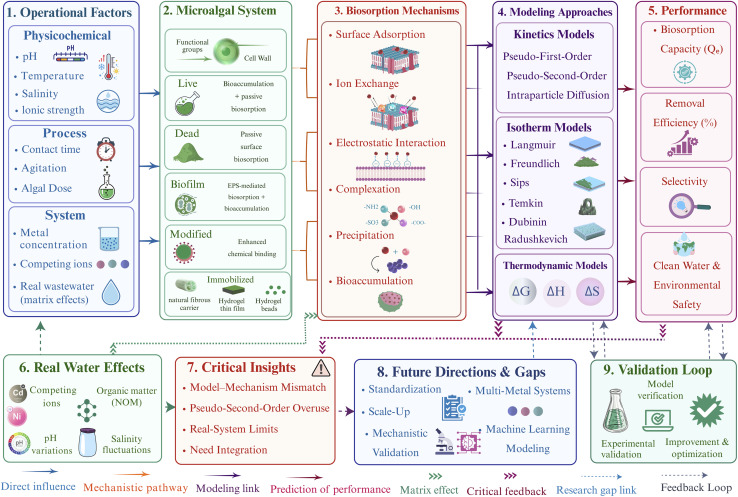
Mechanistic modeling integrated framework for microalgal heavy metal removal. The framework links operational conditions and wastewater matrix characteristics with microalgal biomass properties, surface chemistry, transport processes, and interacting biosorption mechanisms. These processes are connected to kinetic, equilibrium, thermodynamic, and predictive modeling approaches to estimate treatment performance. A validation loop links model predictions with experimental measurements, enabling iterative model refinement and process optimization under increasingly realistic wastewater conditions.

Despite advances in mechanistic understanding, several factors continue to limit the development of reliable predictive frameworks. First, substantial variability in microalgal species, cultivation conditions, biomass composition, and post-harvest processing produces major differences in biosorption performance, partly because these factors alter cell-wall architecture, surface chemistry, and functional-group distribution.^128^ Consequently, models developed using one biomass type or experimental configuration may have limited transferability to other species or preparation conditions. Similar limitations have been identified more broadly in bio-adsorbent research, where studies have often focused on single-metal systems and one-factor-at-a-time optimization, while desorption and regeneration of spent adsorbents remain comparatively underexplored, limiting the development of efficient and scalable treatment strategies.^192^ Second, most experimental datasets are generated under simplified conditions and therefore provide limited information about nonlinear interactions in real wastewater systems. Competitive binding among multiple metals, interactions with organic contaminants, changes in ionic strength, and shifts in metal speciation are rarely represented simultaneously in conventional datasets or models.^193^ This limits the ability of existing models to predict performance outside the conditions under which they were developed. Third, biomass state introduces an additional source of model uncertainty. Living, non-viable, immobilized, chemically modified, and biofilm-based systems differ in surface chemistry, biological activity, transport behavior, and regeneration potential.^194^ Although immobilization can improve biomass recovery and operational stability, the immobilization matrix may introduce additional diffusion resistance and spatial heterogeneity that are not adequately represented in many conventional modeling approaches.^195^ Collectively, these limitations reveal that the principal challenge is not simply the absence of additional adsorption models, but the lack of transferable frameworks that can connect mechanistic information with predictive performance across different biomass types, wastewater matrices, and operating conditions. A major research gap therefore lies in developing validated mechanistic predictive frameworks that integrate interfacial chemistry, transport behavior, biomass characteristics, and environmental variability rather than treating these factors as independent variables, Fig. 5.

## 9. Toward predictive biosorption models and sustainable application

Future progress in microalgal biosorption will require a transition from empirical performance description toward mechanism-informed predictive frameworks. Such frameworks should connect metal speciation, biomass surface chemistry, binding-site characteristics, transport behavior, and operational conditions with measurable treatment outcomes. Rather than relying solely on conventional equilibrium or kinetic fitting, future models should integrate mechanistic information with data-driven approaches capable of representing nonlinear interactions and system variability. Hybrid mechanistic–machine-learning approaches may provide a particularly promising strategy because they can combine the interpretability of physicochemical models with the capacity of data-driven methods to capture complex relationships.^196^ However, predictive performance alone will not establish practical applicability. Models and biosorption systems must be evaluated using environmentally relevant wastewater matrices and, where possible, under pilot- or continuous-flow conditions to determine their transferability beyond optimized batch experiments.^197^

Standardized reporting of biomass origin, cultivation conditions, pretreatment, surface characterization, wastewater composition, competing ions, operating conditions, and regeneration procedures would also improve cross-study comparability and facilitate the development of more reliable datasets. Future research should also evaluate biosorption within broader resource-recovery and circular-economy frameworks. The integration of metal removal with biomass valorization and the recovery of valuable metals could improve the overall sustainability and economic attractiveness of microalgal treatment systems. In parallel, advances in engineered membrane and porous-material platforms suggest a promising design direction for overcoming some of the recovery, recyclability, and processability limitations associated with dispersed biosorbents. Although such materials are not directly equivalent to microalgal biomass, their tunable structural features and engineered transport properties could inspire the development of hybrid microalgal material platforms that combine the biological metal-binding functionality of microalgae with improved structural stability, pollutant accessibility, separation, and reuse. Such integrated systems may provide a pathway toward more controllable and continuously operated biosorption processes, particularly when combined with predictive modeling and mechanistic characterization.^183^ Particular attention should be given to long-term biosorbent stability, regeneration efficiency, capacity retention during repeated cycles, and performance under continuous operation.^189^ Finally, techno-economic assessment, life-cycle analysis, and compliance with environmental discharge standards should be incorporated into the evaluation of future biosorption technologies to determine whether laboratory-scale performance can be translated into technically and economically viable treatment systems. Overall, advancing microalgal biosorption toward practical application will require the integration of mechanistic understanding, predictive modeling, realistic process validation, and techno-economic and environmental assessment rather than further optimization based solely on removal efficiency under idealized laboratory conditions.

## 10. Conclusion

Microalgal biosorption represents a promising and potentially sustainable strategy for heavy-metal removal from contaminated aquatic systems. However, its performance cannot be attributed to a single universal mechanism or evaluated solely on the basis of removal efficiencies obtained under optimized laboratory conditions. Metal uptake is governed by interacting processes, including ion exchange, surface complexation, electrostatic interactions, precipitation, and, in living systems, intracellular uptake. The relative contribution of these processes depends on biomass characteristics, metal speciation, surface chemistry, and environmental conditions. From the authors' perspective, one of the major challenges facing the field is the persistent gap between the apparent performance of microalgal biosorbents under simplified laboratory conditions and their behavior in complex real-world wastewater systems. Conventional kinetic, equilibrium, and thermodynamic models often provide useful empirical descriptions but have limited capacity to represent heterogeneous biomass surfaces, multimetal competition, dynamic wastewater matrices, and transport limitations. Similarly, operational variables such as pH, ionic strength, competing ions, and biomass state are frequently treated as independent factors despite their nonlinear and interdependent effects on biosorption performance. We therefore envisage that the future development of microalgal biosorption should move beyond isolated parameter optimization and capacity-centered evaluation toward mechanism-informed and predictive approaches. Hybrid frameworks integrating surface chemistry, metal speciation, mass transfer, biomass properties, and data-driven prediction could improve model transferability and reduce the gap between laboratory observations and real-world performance. However, such advances must be accompanied by validation in complex wastewater matrices, continuous- or pilot-scale systems, and systematic assessment of regeneration, long-term stability, techno-economic feasibility, and environmental sustainability. In our view, addressing these interconnected challenges will be essential for translating the considerable biosorption potential of microalgal biomass into reliable, scalable, and environmentally sustainable water-treatment technologies. Future progress will depend not only on achieving higher removal capacities but also on developing biosorbent systems whose mechanisms are well understood, performance is predictable under realistic conditions, and operation is compatible with regeneration, resource recovery, and long-term industrial application.

## Author contributions

Heba M. Youssef contributed to conceptualization, literature synthesis, critical analysis of the reviewed studies, framework development, visualization (including all figures and graphical design), and writing of the original draft. Mohamed S. Abd Elhameed and Fatma Mohamed contributed to critical review, refinement of the manuscript, and editing. All authors contributed to the interpretation of the literature, critical discussion of the content, manuscript revision, and approved the final version.

## Conflicts of interest

The authors declare that they have no known competing financial interests or personal relationships that could have appeared to influence the work reported in this paper.

## Data Availability

Data sharing is not applicable to this article as no new data were created or analyzed in this study.
